# 2414: Reperfusion strategies when non-stemi is misclassified as stemi myocardial infarction

**DOI:** 10.1017/cts.2017.272

**Published:** 2018-05-10

**Authors:** Pablo I. Altieri, Alejandro Figueroa, Ismael Valle, Orlando Arce, Brigida Colon, Hector Banchs, Pablo Altieri

**Affiliations:** University of Puerto Rico-Medical Sciences Campus, San Juan, Puerto Rico

## Abstract

OBJECTIVES/SPECIFIC AIMS: A retrospective analysis was done at the Cardiovascular Center to evaluate reperfusion strategies, including stemi infarcts and non-stemi classified as stemi in a period of 2 years. METHODS/STUDY POPULATION: Review the records of stemi infarcts in a period of 2 years. RESULTS/ANTICIPATED RESULTS: In total, 101 cases were classified as stemi, but after strict analysis (time wise) 24 cases were non-stemi; 47% had inferior myocardial infarction and 38% an anterior myocardial infarction with a mean age of 65 years. All cases were immediately catheterized. Although the non-stemi, classified originally as stemi did not meet the time limit (<2 h) for cath. The stemi group (77 P.) 58 P. had angioplasty with stent implantation. 19 P. had an EF of 45% and remained that way during follow up. The rest of the P. the EF went up to 50% or more. The non-stemi group (24 P.) had angioplasty with stent implantation. The EF remained around 40% during follow up, which was the EF on admission. Fibrinolysis was given erratically. No changes were seen in the EF on follow up in the fibrinolytic group. DISCUSSION/SIGNIFICANCE OF IMPACT: This shows the importance of classifying the P. well between stemi and non-stemi. The time frame to catheterization should be kept as strict as possible, due to transmural infarcts, and catheterized in <2 hours to avoid deterioration of the left ventricular function and its consequences. This strict classification will save money to the institution when emergency catheterization is avoided in the non-stemi group.

